# Characterization and Regulation of the Acetolactate Synthase Genes Involved in Acetoin Biosynthesis in *Acetobacter pasteurianus*

**DOI:** 10.3390/foods10051013

**Published:** 2021-05-06

**Authors:** Jingyi Zhao, Zhe Meng, Xiaolong Ma, Shumei Zhao, Yang An, Zijun Xiao

**Affiliations:** Center for Bioengineering and Biotechnology, College of Chemical Engineering, China University of Petroleum (East China), 66 Changjiang West Road, Huangdao District, Qingdao 266580, China; jyzhao@upc.edu.cn (J.Z.); S18030117@s.upc.edu.cn (Z.M.); Z17030374@s.upc.edu.cn (X.M.); Z15030319@s.upc.edu.cn (S.Z.); Z20030141@s.upc.edu.cn (Y.A.)

**Keywords:** acetoin, *Acetobacter pasteurianus*, acetolactate synthase, LysR-type transcriptional activator, benzoylformate decarboxylase, quantitative reverse transcription PCR

## Abstract

Acetoin is an important aroma-active chemical in cereal vinegars. *Acetobacter pasteurianus* was reported to make a significant contribution to acetoin generation in cereal vinegars. However, the related acetoin biosynthesis mechanism was largely unknown. Two annotated acetolactate synthase (ALS) genes of *A. pasteurianus* were investigated in this study to analyze their functions and regulatory mechanisms. Heterologous expression in *Escherichia coli* revealed that only AlsS1 exhibited ALS activity and had the optimal activity at 55 °C and pH 6.5. Two *alsS*-defective mutants of *A. pasteurianus* CICC 22518 were constructed, and their acetoin yields were both reduced, suggesting that two *alsS* genes participated in acetoin biosynthesis. A total 79.1% decrease in acetoin yield in the *alsS1*-defective mutant revealed that *alsS1* took a major role. The regulator gene *alsR* disruptant was constructed to analyze the regulation effect. The decline of the acetoin yield and down-regulation of the *alsD* and *alsS1* gene transcriptions were detected, but the *alsS2* gene transcription was not affected. Acetoin was an important metabolite of lactate catabolism in *A. pasteurianus*. The coexistence of two *alsS* genes can help strains rapidly and securely assimilate lactate to deal with the lactate pressure in a vinegar brewing environment, which represented a new genetic mode of acetoin production in bacteria.

## 1. Introduction

Acetoin (3-hydroxy-2-butanone) is an important bio-based platform chemical that can be generated by many bacteria using different carbon substrates such as glucose, sucrose, glycerol, cellulose, acetate, carbon dioxide, and so on [1,2,3]. This compound has a pleasant yogurt odor and a fatty butter taste, so it has been found in a variety of fermented foods and beverages [4]. In our previous study, we detected acetoin in many types of vinegars, particularly in the solid-state fermentation (SSF) vinegars [5]. Chinese vinegars are commonly produced through the SSF process with various cereals as raw materials [6,7]. Complex microbiota in the brewing starter (termed as vinegar *Pei*) participate in this fermentative process [7,8] and generate acetic acid, lactic acid, amino acids, aldehydes, alcohols, ketones, esters, phenols, heterocyclic compounds, and so on, which enable the cereal vinegars to have better flavors and tastes [6,9]. In some Chinese vinegars, acetoin can reach the highest concentration of 4.0 g/L [10] and becomes one kind of the major volatile compounds. In addition, acetoin was identified as the precursor of 2,3,5,6-tetramethylpyrazine (TMP) [5], a bioactive molecule in Chinese cereal vinegars that had dietotherapy functions for cardiovascular and cerebrovascular health [11]. Thus, improving the acetoin production in the SSF process will help to increase the TMP concentration in vinegars and enhance the quality of vinegars.

Because acetoin is regarded as an important aroma-active chemical and bioactive molecule precursor, its biosynthesis mechanism in cereal vinegars has attracted much attention. Recent investigations found that *Acetobacter pasteurianus* and some *Lactobacillus* species potentially made significant contributions to the accumulation of acetoin in cereal vinegars [12,13]. *A. pasteurianus* is one kind of important acetic acid bacteria (AAB) that is widely applied in industrial vinegar production. *A. pasteurianus* strains have been identified to generate acetoin since 1959 [14], but those enzymes involved in acetoin metabolism have not been investigated. In addition, *A. pasteurianus* strains can efficiently convert ethanol to acetic acid, but consume glucose slowly [13]. Glucose is the preferred substrate for most acetoin-producing bacteria, suggesting that different acetoin metabolic mechanisms may occur in *A. pasteurianus*.

In the reported acetoin-producing microbes, two key enzymes are found to be responsible for pyruvate bio-conversion to acetoin [1]. The acetolactate synthase catalyzes the condensation of two molecules of pyruvate to generate one molecule of α-acetolactate and the acetolactate decarboxylase (ALDC) subsequently decarboxylates α-acetolactate to form acetoin. The acetolactate synthase (EC 2.2.1.6) includes two types: the anabolic α-acetolactate synthase (more commonly termed as acetohydroxy acid synthase, AHAS) and the catabolic α-acetolactate synthase (ALS in this study) [4]. AHAS contains two subunits, the large subunit and the small subunit, and it converts pyruvate to α-acetolactate with very low efficiency. ALS can convert pyruvate to α-acetolactate with high efficiency, and it coexists with ALDCs in almost all known species. ALS and ALDC are encoded by *alsS* and *alsD*, respectively, which are located in an acetoin operon, occasionally along with the 2,3-butanediol dehydrogenase gene *bdh*. Additionally, these genes are regulated by the LysR-type transcriptional activator gene *alsR*, which is often present upstream of the acetoin operon but transcribes divergently.

Based on these basic understandings, we analyzed the genomic sequences of different *A. pasteurianus* strains available in GenBank and looked for the genes possibly involved in acetoin biosynthesis. As a result, we found a gene cluster containing the potential *alsR*, *alsD*, and *alsS* (here termed as *alsS1*) genes in the chromosome. Interestingly, another gene (here termed as *alsS2*) encoding a putative ALS was located downstream of this *alsRDS1* gene cluster. The presence of two ALS-encoding genes in tandem was conserved in all sequenced *A. pasteurianus* stains, which was quite different from the genetic arrangements in previously reported acetoin operons [1]. Whether *alsS1* and *alsS2* both participated in acetoin biosynthesis of *A. pasteurianus* required further investigations.

In the present study, we aimed to investigate the functions and regulatory mechanisms of the *alsS1* and *alsS2* genes. The *alsS1* and *alsS2* genes were expressed in *Escherichia coli* to evaluate their enzymatic activities. Meanwhile, these two genes were separately knocked out to construct *A. pasteurianus* mutants and assess their physiological functions in vivo. In addition, the *alsR* gene was knocked out to analyze the regulatory effects on *alsS1* and *alsS2* by quantitative reverse transcription PCR (qRT-PCR). This study will help us to gain a better understanding of these two novel genes and the acetoin biosynthesis mechanism in *A. pasteurianus*.

## 2. Materials and Methods

### 2.1. Strains and Plasmids

The bacterial strains and plasmids used in this study were described in Table 1. *A. pasteurianus* CICC 22518 was purchased from the China Center of Industrial Culture Collection. *E. coli* BL21(DE3), *E. coli* DH5α, and *E. coli* HB101(pRK2013) were used for gene expression, cloning, and conjugation, respectively.

### 2.2. Heterologous Expression

The genomic DNA of *A. pasteurianus* CICC 22518 was extracted and used as the template DNA for PCR. The *alsS1* and *alsS2* genes were individually amplified using the primers 28alsS1F/R and 22alsS2F/R and then digested by *Nde*I and *Xho*I. The primers used in this study were listed in Table 2. The *alsS1* gene was ligated to pET-28a(+) and transformed to *E. coli* DH5α, resulting in plasmid pET28S1. The *alsS2* gene was ligated to pET-22b(+) and transformed to *E. coli* DH5α, resulting in plasmid pET22S2. After verification by DNA sequencing, two plasmids were separately transformed to *E. coli* BL21(DE3), resulting in *E. coli* BL21-S1 and BL21-S2 for protein expression.

*E. coli* BL21-S1 was incubated in LB medium containing 50 mg/L kanamycin at 37 °C until the optical density at 600 nm (OD_600_) reached 0.4. A total of 0.6 mM isopropyl-β-d-thiogalactopyranoside (IPTG) was added in the culture, which was incubated at 37 °C for another 7 h. *E. coli* BL21-S2 was incubated in LB medium containing 100 mg/L ampicillin at 37 °C until OD_600_ reached 0.6. A total of 0.5 mM IPTG was added in the culture, which was incubated at 25 °C for 10 h.

*E. coli* cells were collected by centrifugation and suspended in 0.1 M sodium phosphate buffer (pH 6.5). After cell disruption by sonication and centrifugation, the crude extracts were loaded on the nickel affinity column HisTrap FF crude (GE Healthcare Bio-Sciences AB, Uppsala, Sweden) and subsequently desalted using the HiTrap desalting column (GE Healthcare Bio-Sciences AB, Uppsala, Sweden). Finally, the purified recombinant proteins were dissolved in 0.1 M sodium phosphate buffer (pH 6.5) at 4 °C. The protein concentrations were determined by TaKaRa Bradford Protein Assay Kit (TaKaRa, Dalian, China). The protein size and purity were measured by SDS-PAGE.

### 2.3. ALS Activity Assay

ALS activity was determined by monitoring the amount of acetoin according to the creatinine colorimetric assay method. Briefly, 20 μL of the purified protein was added into 480 μL of the reaction buffer that contained 100 mM sodium pyruvate, 0.5 mM magnesium chloride, 1 mM thiamine pyrophosphate (TPP) in 100 mM sodium phosphate buffer (pH 6.5), and then incubated at 55 °C for 20 min. A total of 20 μL of 50% sulfuric acid was added into the mixture and incubated at 55 °C for 30 min. Finally, 250 μL of 0.5% creatine and 250 μL of 5% α-naphthol were added into the reaction mixture and incubated at 37 °C for 30 min. Absorbance at 520 nm was measured to quantify the amount of acetoin in the mixture. One unit of ALS activity was defined as the generation of 1 μmol acetoin in 1 min.

The effect of temperature on enzymatic activity was determined at temperatures ranging from 25 to 70 °C. The effect of pH on enzymatic activity was determined in different buffers, including sodium acetate buffer (pH 4, 5, and 6), sodium phosphate buffer (pH 6, 6.5, 7, and 8), and glycine-sodium hydroxide buffer (pH 8, 9, and 10). The effects of branched-chain amino acids, EDTA, NAD^+^, NADH, NADP^+^, NADPH, and metal ions on enzymatic activity were determined at the optimal temperature and pH.

The kinetic parameters of AlsS1 were measured using different concentrations of sodium pyruvate as substrate. The values of *K*_m_ and *V*_max_ were obtained based on extracting the slope and intercept from a double reciprocal Lineweaver–Burk plot.

### 2.4. Benzoylformate Decarboxylase (BFD) Activity Assay

The BFD activity was determined by monitoring the generation of benzaldehyde. A total of 200 μL of the AlsS2 protein was added into 4.80 mL of the reaction buffer that contained 10 mM benzoylformate, 0.5 mM magnesium chloride, 1 mM TPP in 100 mM sodium phosphate buffer (pH 7.0), and then incubated at 37 °C for 10 h. One gram of dichloromethane was added to the reaction mixture. After vigorously blending, the organic phase was collected and detected by gas chromatography (GC) technique with a 30-m HP-5 capillary column (HP Agilent, Gainesville, GA, USA). The operation conditions were as follows: N_2_ was used as the carrier gas at a flow rate of 2.3 mL/min; the injector temperature was 280 °C, and the detector temperature was 300 °C; the column temperature was maintained at 50 °C for 2 min, and subsequently increased to 280 °C at a rate of 10 °C/min. The benzaldehyde concentration was determined by using a calibration curve, and *n*-hexanol was used as the internal standard. One unit of BFD activity was defined as the generation of 1 μmol benzaldehyde in 1 min.

### 2.5. Construction of A. pasteurianus Mutants

The markerless deletion vector pKOS6b [15] containing the *codAB* genes responsible for 5-fluorocytosine (FC) sensitivity was used to construct the gene deletion plasmid. The upstream and downstream regions of the target genes (*alsS1*, *alsS2*, and *alsR*) were amplified by PCR using primers LS1-dup1, S1-ddw1, S1-dup2, LS1-ddw2, LS2-dup1, S2-ddw1, S2-dup2, LS2-ddw2, L-R-dup1, L-R-ddw1, L-R-dup2, and L-R-ddw2. The PCR products were cloned into the *Hin*dIII-*Sma*I-pretreated pKOS6b using Trelief™ SoSoo Cloning Kit Ver.2 (Beijing TsingKe Biotech, Beijing, China) according to the manufacturer’s instructions, and then transformed into *E. coli* DH5α. The recombinant plasmids pKOS6b-ΔalsS1, pKOS6b-ΔalsS2, and pKOS6b-ΔalsR were obtained and verified by DNA sequencing.

Triparental mating was carried out to construct the target gene defective mutants of *A. pasteurianus* according to the previously reported method with minor modifications [15]. *E. coli* DH5α containing the target gene deletion plasmid and *E. coli* HB101(pRK2013) were used as the donor strain and helper strain, respectively. *E. coli* strains were grown in LB medium containing kanamycin at 37 °C until OD_600_ reached 1.0. *A. pasteurianus* CICC 22518 was used as the acceptor strain and grown in YPGE medium (polypeptone 5 g/L, yeast extract 5 g/L, glycerol 5 g/L, and ethanol 40 mL/L) at 30 °C until OD_600_ reached 1.0. *E. coli* donor strain, helper strain, and A. *pasteurianus* CICC 22518 were mixed at a ratio of 1:1:4, and subjected to centrifugation. The cell pellets were collected and suspended in YPG medium (polypeptone 5 g/L, yeast extract 5 g/L and glycerol 5 g/L). A total of 100 μL of the mixture was dropped on YPG plates and then incubated at 30 °C for 24 h. After a series of dilutions, the culture was plated on YPGE plates containing 50 mg/L kanamycin and 50 mg/L chloramphenicol. After the transconjugants grew, colony PCR was performed to determine whether the target gene deletion plasmid had integrated into the chromosome of *A. pasteurianus* by the first cross-over homologous recombination. Subsequently, the positive clones were inoculated in YPGE medium and plated on YPGE plates containing 60 mg/L FC. When the survival clones grew, they were selected to perform colony PCR and determine if the second cross-over homologous recombination occurred. Those that had the desired gene deletions were confirmed by DNA sequencing, resulting in the *A. pasteurianus* mutants: 22518-ΔalsS1, 22518-ΔalsS2, and 22518-ΔalsR.

### 2.6. Comparative Analyses of A. pasteurianus Wild Strain and Mutants

*A. pasteurianus* strains were inoculated in a YPG medium containing 2% ethanol at 30 °C with vigorous agitation to analyze acetic acid production and ethanol consumption. Strain growths were periodically measured by a spectrophotometer at 600 nm. The acetic acid and ethanol concentrations in culture broths were determined by GC technique with the ZKAT-LZP 930.2a column (0.32 mm × 30 m) (ATEO, Lanzhou, China). A total of 1 mL of the culture broth was subjected to centrifugation, and the supernatants were collected and mixed with 0.4 g of 0.5% propanol. The operation conditions were as follows: N_2_ was used as the carrier gas at a flow rate of 0.6 mL/min; the injector and detector temperature were 230 °C; the column temperature was maintained at 50 °C for 7 min, and subsequently increased to 230 °C at a rate of 5 °C/min. The concentrations of acetic acid and ethanol were determined by using calibration curves, and propanol was used as the internal standard.

For the purpose of analyzing acetoin metabolism, *A. pasteurianus* strains were inoculated in LYSE medium [L-lactic acid (TCI, Japan) 10 g/L, yeast extract 10 g/L, soy peptone 5 g/L, ethanol 10 mL/L, pH 4.5] at 30 °C with vigorous agitation. The acetoin concentrations in culture broths were measured by the GC technique according to the previously reported method [16]. The L-lactic acid concentrations in culture broths were determined by an enzymatic-membrane assay on the Biosensors Analyzer S-10 (Sieman, Shenzhen, China) according to the manufacturer’s instruction.

### 2.7. RNA Isolation and qRT-PCR Analysis

*A. pasteurianus* strains were inoculated in LYSE medium at 30 °C with vigorous agitation for 12 h. RNA was extracted using SV Total RNA Isolation System (Promega, Madison, WI, USA), and then reverse-transcribed using PrimeScript™ RT Reagent Kit with gDNA Eraser (Perfect Real Time) (TaKaRa, Dalian, China) and four specific primers alsDdw1, alsS1dw1, alsS2dw1, and gyrAdw1. Real-time PCR assays were performed in triplicates on the Applied Biosystems ABI 7500 Real-time PCR System using TB Green^®^ *Premix Ex Taq*™ II (Tli RNaseH Plus) (TaKaRa, Dalian, China). The relative transcriptional levels of target genes were calculated using the 2^−ΔΔCt^ method, and the *gyrA* gene was used as the reference gene.

### 2.8. Statistical Analysis

All experiments were repeated at least three times. Data were means ± standard deviations (*n* = 3), and Microsoft Excel 2016 (Microsoft Corp, Redmond, WA, USA) was used for the statistical testing. *p* < 0.05 was considered statistically significant.

## 3. Results

### 3.1. Bioinformatic Analyses

**Figure 1:**
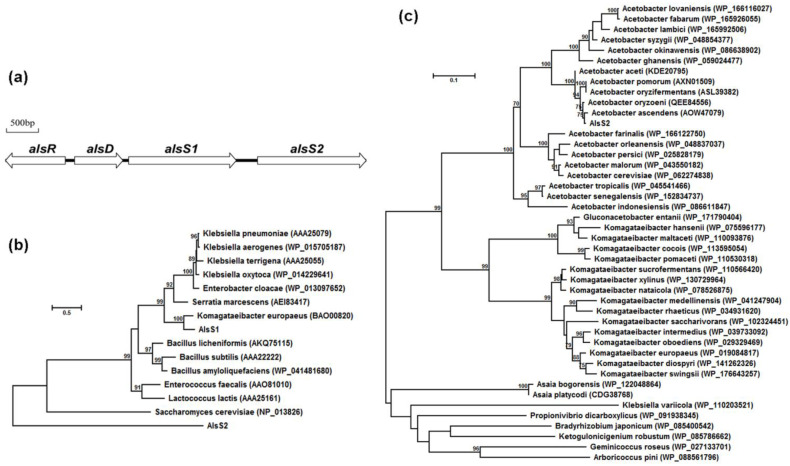
The gene cluster related to acetoin biosynthesis in *A. pasteurianus* CICC 22518 (**a**), phylogenetic tree of AlsS1, AlsS2, and reported ALSs involved in acetoin biosynthesis (**b**), phylogenetic tree of AlsS2 and similar analogs in GenBank (**c**). The reconstruction was computed by the maximum likelihood method with bootstrapping test (1000 replicates) using MEGA X. GenBank accession numbers of the reference sequences were labeled in parentheses.

Complete genomic sequences of 14 *A. pasteurianus* strains had been available in GenBank databases. Based on the annotated gene information, we found a gene cluster containing the potential *alsR*, *alsD*, *alsS1*, and *alsS2* genes in the chromosomal sequences (Figure 1a). The *alsD*, *alsS1*, and *alsS2* genes were transcribed in the same orientation. The *alsR* gene was located upstream of *alsD* with opposite transcriptional orientation. The existence of two *alsS* genes in tandem in the chromosome was conserved in all sequenced *A. pasteurianus* strains, which was quite different from the genetic arrangements in the reported acetoin operons that all contained only one *alsS* gene [1].

The *alsS1* (1689 bp) and *alsS2* (1695 bp) genes had similar size but they shared only 23.0% amino acid sequence identities. Phylogenetic analyses of the two AlsS proteins and other reported ALSs involved in acetoin biosynthesis were performed using MEGA X (Figure 1b) [17]. The AlsS1 protein shared 53.9–76.9% amino acid sequence identities with the ALSs in Gram-negative bacteria, 46.9–52.2% amino acid sequence identities with the ALSs in Gram-positive bacteria, and only 25.8% amino acid sequence identities with the ALS of *Saccharomyces cerevisiae*. So AlsS1 was more similar to the ALSs in Gram-negative bacteria. However, the AlsS2 protein shared only 21.9–32.0% amino acid sequence identities with these reported ALSs involved in acetoin biosynthesis, indicating that the AlsS2 protein was quite different.

Alignment of AlsS2 and other similar analogs in GenBank was carried out by BlastP. It was found that AlsS2 was more similar to the annotated ALSs or TPP-binding proteins originating from different *Acetobacter* and *Komagataeibacter* species (Figure 1c). In particular, AlsS2 shared 83.2–99.5% amino acid sequence identities with the annotated ALSs in different *Acetobacter* species. In some *A. pasteurianus* strains, AlsS2 was also annotated as BFD. However, it displayed only 33.5% amino acid sequence identities with the MdlC protein of *Pseudomonas putida* ATCC 12633 [18], a representative BFD.

### 3.2. Heterologous Expression and Enzymatic Analyses

**Figure 2:**
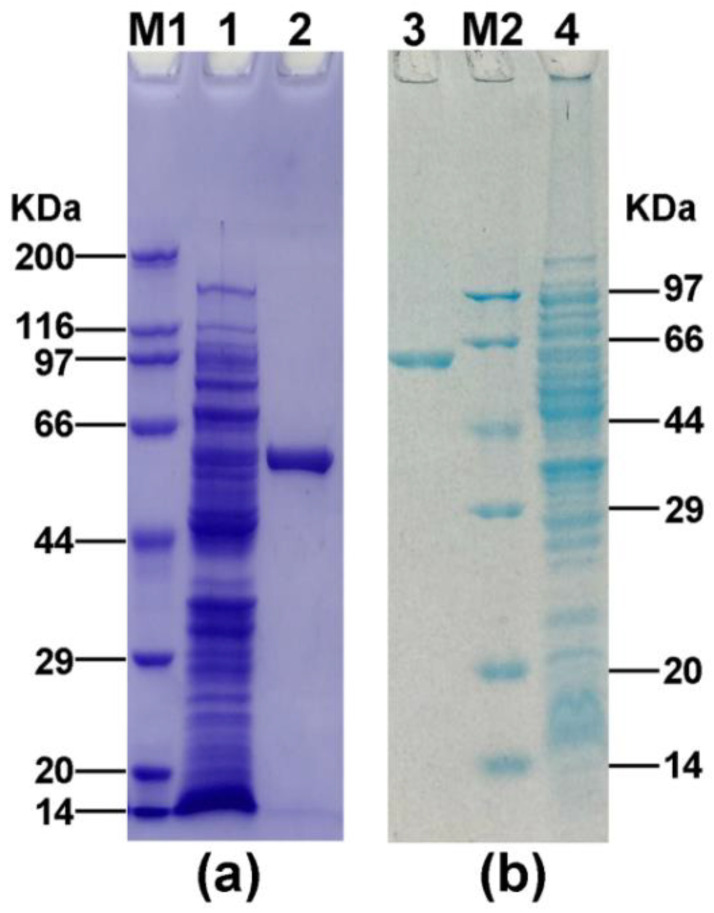
SDS-PAGE analysis of AlsS1 (**a**) and AlsS2 (**b**) expressed in *E. coli*. Lane M1, premixed protein marker (Broad) (TaKaRa, Dalian, China); lane 1, induced *E. coli* BL21-S1; lane 2, purified AlsS1; lane 3, purified AlsS2; lane M2, premixed protein marker (Low) (TaKaRa, Dalian, China); lane 4, induced *E. coli* BL21-S2.

Considering the novelty of the two AlsS proteins of *A. pasteurianus*, we attempted to analyze their enzymatic activities by heterologous expression. *A. pasteurianus* CICC 22518 used in this study was originally isolated from vinegar *Pei*. We had sequenced its genomic sequences and deposited them in the GenBank database (accession numbers CP039845-CP039847). The *alsS1* and *alsS2* genes of *A. pasteurianus* CICC 22518 were amplified by PCR, cloned into expression vectors, and then transformed into *E. coli*. After protein expression and purification, two His-tagged AlsS proteins were determined to have the desirable sizes (about 59 kDa) by SDS-PAGE (Figure 2). The AlsS1 concentration was 26.5 μg/mL, and the AlsS2 concentration was 24.5 μg/mL.

**Figure 3:**
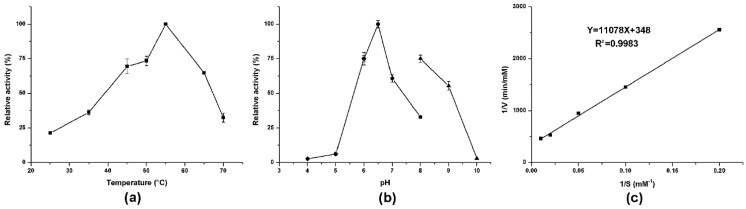
Effects of different temperatures (**a**) and pH (**b**) on the enzymatic activity of AlsS1 and Lineweaver–Burk plot of AlsS1 (**c**). The pH effect was measured in sodium acetate buffer (diamond), sodium phosphate buffer (circle), or glycine-sodium hydroxide buffer (uptriangle). Data were expressed as mean ± standard errors of three replicates.

Enzymatic activity analyses revealed that AlsS1 had suitable ALS activity. The optimal temperature and pH were 55 °C and pH 6.5, respectively (Figure 3a,b). The metal ions (Na^+^ and K^+^), four cofactors (NAD^+^, NADH, NADP^+^, and NADPH), and three branched-chain amino acids (Leu, Ile, and Val) had no significant effects on the enzymatic activity of AlsS1 (Table 3). EDTA can dramatically reduce the AlsS1 activity. The kinetic parameters of AlsS1 were measured using sodium pyruvate as the substrate under the optimal temperature and pH. The catalytic rate by AlsS1 followed Michaelis–Menten kinetics. Based on the Lineweaver–Burk plot of the initial rates plotted against the concentrations of sodium pyruvate, the *K*_m_ and *V*_max_ values were calculated for 31.8 mM and 2.9 μM/min, respectively (Figure 3c).

The ALS activity was not detected for AlsS2. Considering the test sensitivity, we tried to measure the acetoin amount in the enzymatic reaction mixtures by GC, but no acetoin was detected. In addition, we changed sodium pyruvate to benzoylformate as the substrate to analyze the enzymatic activity of AlsS2. Benzaldehyde was detected in the reaction mixture (Supplementary Materials, Figure S1) by GC analysis, indicating that AlsS2 had the BFD activity (2.74 U/mg protein).

### 3.3. Metabolite Profiles of A. pasteurianus CICC 22518 and the Two alsS-Defective Mutants

In order to examine the functionality of the *alsS1* and *alsS2* genes in *A. pasteurianus*, we attempted to construct two *alsS*-defective mutants. Plasmid pKOS6b [15], a markerless gene deletion vector previously used in *Gluconobacter* strains, was applied for gene knockout in this study. By virtue of the in-fusion cloning technique, we constructed two recombination plasmids: pKOS6b-ΔalsS1 (containing 1.9 kb upstream regions and 1.8 kb downstream regions of *alsS1*) and pKOS6b-ΔalsS2 (containing 1.9 kb upstream regions and 1.7 kb downstream regions of *alsS2*). Two plasmids were individually transferred to *A. pasteurianus* CICC 22518 by conjugation. After twice clone screenings by kanamycin or FC resistance, *A. pasteurianus* 22518-ΔalsS1 and 22518-ΔalsS2 were obtained. Colony PCR of the target regions were performed, and sequencing analysis revealed that the *alsS1* and *alsS2* gene were deleted as expected, respectively.

**Figure 4:**
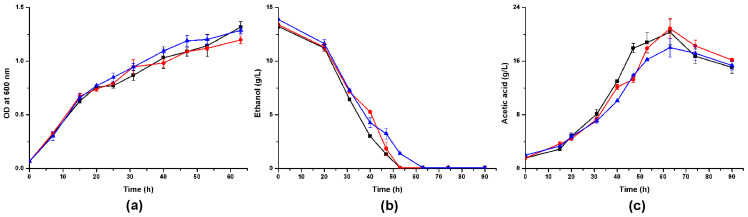
Time course of the cell growth (**a**), ethanol consumption (**b**), and acetic acid production (**c**) in YPG media containing 2% ethanol by *A. pasteurianus* CICC 22518 (square), 22518-ΔalsS1 (circle), and 22518-ΔalsS2 (uptriangle). Data were expressed as mean ± standard errors of three replicates.

We cultivated *A. pasteurianus* CICC 22518 and these two *alsS*-defective mutants in different media and measured their cell growths and metabolites. When these strains were inoculated in YPG media containing 2% ethanol, they all grew well (Figure 4). *A. pasteurianus* 22518-ΔalsS2 grew a little faster than CICC 22518 and 22518-ΔalsS1 (*p* < 0.05). Ethanol consumption rate of *A. pasteurianus* 22518-ΔalsS2 was a little slower than those of CICC 22518 and 22518-ΔalsS1 (*p* < 0.05). Acetic acid productions of three strains had no significant differences (*p* > 0.05). However, the acetoin yields in culture broths were undetectable by the GC technique. Therefore, we tried to change the carbon source in the medium to investigate the acetoin metabolism in *A. pasteurianus*.

**Figure 5:**
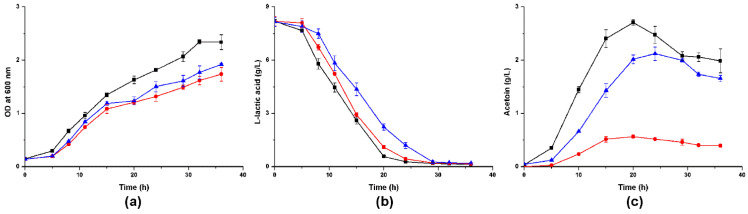
Time course of the cell growth (**a**), lactic acid consumption (**b**), and acetoin production (**c**) in LYSE media by *A. pasteurianus* CICC 22518 (square), 22518-ΔalsS1 (circle), and 22518-ΔalsS2 (uptriangle). Data were expressed as mean ± standard errors of three replicates.

Given that lactic acid can stimulate *Acetobacter* to produce acetoin [14,19,20], we inoculated these *A. pasteurianus* strains in LYSE media and found that acetoin production was activated. *A. pasteurianus* can generate acetoin during the logarithmic phase. Compared with the wild strain, the growth rates and L-lactic acid assimilation rates of the two mutants were slower (Figure 5a,b), and both mutants reduced their acetoin yields (*p* < 0.05). Nearly 79.1% decrease was detected in *A. pasteurianus* 22518-ΔalsS1, and 21.6% decline was detected in 22518-ΔalsS2 (Figure 5c).

### 3.4. Regulation Effect by alsR

The LysR-type regulator gene *alsR* was reported to regulate the structural genes *alsD* and *alsS* so as to affect acetoin generation [21]. Given that two *alsS* genes in *A. pasteurianus* were related to acetoin biosynthesis, we aimed to construct the *alsR*-defective mutant to evaluate whether the expressions of these two *alsS* genes were both under the control of *alsR*. The recombinant plasmid pKOS6b-ΔalsR, containing 1.9 kb upstream regions and 1.8 kb downstream regions of *alsR*, was constructed and transferred to *A. pasteurianus* CICC 22518 by conjugation. After two cross-over homologous recombination, the desired transconjugant with the *alsR* gene deletion, *A. pasteurianus* 22518-ΔalsR, was obtained. Colony PCR and sequencing analysis revealed that the *alsR* gene was knocked out.

**Figure 6:**
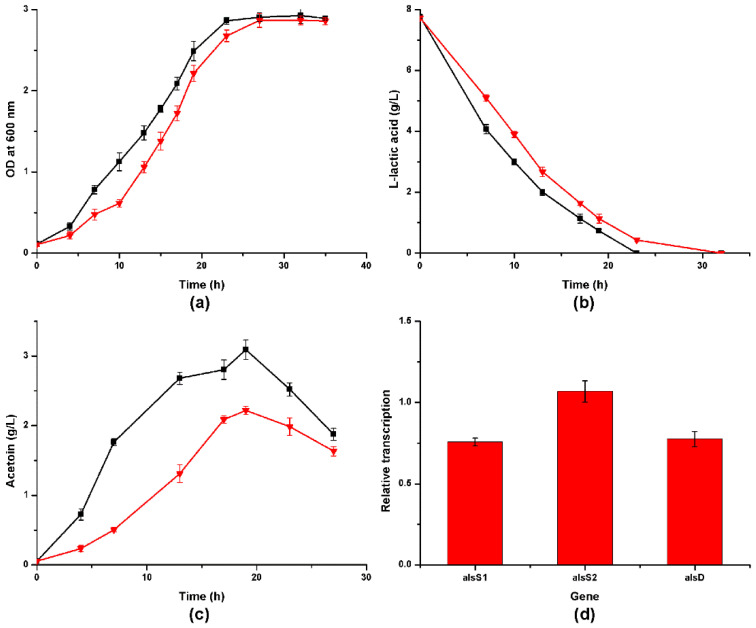
Time course of the cell growth (**a**), lactic acid consumption (**b**), and acetoin production (**c**) by *A. pasteurianus* CICC 22518 (square) and 22518-ΔalsR (downtriangle). Relative transcriptional level of the *alsS1*, *alsS2*, and *alsD* genes of *A. pasteurianus* 22518-ΔalsR to CICC 22518 (**d**). Data were expressed as mean ± standard errors of three replicates.

Compared with the wild strain, *alsR* gene deletion induced the mutant to grow a little slower and yield less acetoin (*p* < 0.05) (Figure 6), about 71.9% of the maximum acetoin yield of *A. pasteurianus* CICC 22518. Subsequently, qRT-PCR was carried out to estimate the transcriptional level of the *alsD*, *alsS1*, and *alsS2* genes. It was revealed that the relative transcriptional levels of the *alsD* and *alsS1* genes were reduced, but the transcription of *alsS2* was unchanged. In detail, the transcriptions of *alsD* and *alsS1* were down-regulated by 22.4% and 24.3%, respectively (Figure 6d).

## 4. Discussion

SSF is an important character of Chinese cereal vinegars and endows the vinegars with better scent and taste. In the recent decade, microbiological investigations were performed in industrial vinegar production in order to clarify the complex microbial fermentative processes and metabolite generation mechanisms [7,8,12]. Because acetoin and TMP are important aroma-active and bioactive molecules, people tried to improve their concentrations in vinegars. Zhang et al. inoculated *Bacillus amyloliquefaciens* producing acetoin and TMP at a high level in the fermentation starter and elevated the acetoin and TMP contents in Baoning bran vinegar [22]. Zhao and Yun screened highly acetoin-producing AAB from the solid substrate of Liangzhou fumigated vinegar in order to apply these strains in the industrial production of vinegars [23]. Chai et al. accelerated the acetoin and TMP production in the vinegar fermentation process through *Lactobacillus* sp.-*A. pasteurianus* joint bioaugmentation [13]. However, detailed investigation on the acetoin metabolic mechanism of AAB in the SSF process was scarce.

*A. pasteurianus* CICC 22518 in this study was isolated from the vinegar *Pei* in a vinegar-producing plant in China. This strain can use ethanol, lactic acid, and glycerol and had a suitable ability to generate acetic acid. Therefore, microbial investigation using this strain can provide suitable instruction for strain improvements in industrial vinegar production. We first sequenced the complete genomic sequence of *A. pasteurianus* CICC 22518 and detected the *alsRDS1S2* gene cluster that was potentially related to acetoin biosynthesis. Based on the sequence alignments and further genomic sequence analysis of different *Acetobacter* species, we found that this gene cluster was present in many *Acetobacter* species, such as *A. ascendens*, *A. cerevisiae*, *A. farinalis*, *A. malorum*, *A. orleanensis*, *A. oryzifermentans*, *A. oryzoeni*, *A. persici*, *A. pomorum*, *A. senegalensis*, and *A. tropicalis*. The existence of two *alsS* genes in tandem seemed to be conserved in most *Acetobacter* species and represented a new mode of acetoin production in bacteria.

Bioinformatic analyses revealed that AlsS1 and AlsS2 had the GDG motif, the Mg^2+^-binding site conserved in TPP-binding proteins, but they shared only 23.0% amino acid sequence identities, indicating that they may originate from different ancestors of TPP-binding protein. Compared with AlsS2, AlsS1 was more similar to the reported ALSs involved in acetoin biosynthesis and showed the highest 76.9% amino acid sequence identities with the ALS protein of *Komagataeibacter europaeus* (Figure 1b). However, it was reported that the *als* disruptant of *K. europaeus* had no effect on acetoin production [19]. Whether the *alsS1* gene played a part in acetoin biosynthesis of *A. pasteurianus* needed detailed investigation.

First, AlsS1 was expressed in *E. coli* and revealed suitable ALS activity. The optimal pH of AlsS1 was pH 6.5, which was similar to most reported ALSs with optimal pH of 6.5–7.0 [24]. The optimal temperature of AlsS1 was 55 °C, indicating that a relatively high temperature can help AlsS1 to better catalyze the substrate, which was in accordance with the muggy vinegar *Pei* environment in the SSF process. The *K*_m_ value of 31.8 mM for pyruvate indicated relatively low activity of AlsS1 in comparison with the ALSs in highly acetoin-producing *Enterococcus faecalis* (1.37 mM), *Bacillus licheniformis* (3.96 mM), and *Klebsiella pneumoniae* (8 mM) [24]. These highly acetoin-producing bacteria can rapidly consume glucose and generate a great deal of acetoin, a neutral molecule, with high efficiency in order to avoid cellular acidification. However, we found that acetoin was not the major metabolite of glucose catabolism in *A. pasteurianus* CICC 22518. When we used glucose (10 g/L) as the carbon source to cultivate CICC 22518, only a trace amount of acetoin (about 0.06 g/L) was generated, indicating relatively poor efficiency of the acetoin biosynthesis pathway in the glucose-containing medium. This phenomenon was in accordance with that there was only a very low amount of reducing sugars existing in vinegar *Pei*.

Subsequently, we constructed the *alsS1*-defective mutant of *A. pasteurianus* CICC 22518 to analyze the influence of *alsS1* deletion on acetoin biosynthesis. When we cultivated *A. pasteurianus* strains in LYSE medium, the *alsS1*-defective mutant reduced 79.1% of the acetoin yield compared with the wild strain, suggesting that *alsS1* took part in acetoin biosynthesis of *A. pasteurianus*, which was in contrast to the *als* gene in *K. europaeus* with no effect on acetoin biosynthesis [19].

Based on sequence alignments, AlsS2 in *A. pasteurianus* was not similar to the reported ALSs involved in acetoin biosynthesis (Figure 1b). AlsS2 showed high amino acid sequence identities with the annotated second ALSs encoded by the *alsRDS1S2* gene cluster in most *Acetobacter* species (Figure 1c). We constructed the *alsS2*-defective mutant of *A. pasteurianus* CICC 22518 and found that the acetoin yield decreased, suggesting that AlsS2 indeed participated in acetoin biosynthesis in *A. pasteurianus*. However, the heterologously expressed AlsS2 protein did not exhibit the ALS activity in vitro, even if we increased the protein amount in the enzymatic assay experiment. Furthermore, we performed the decarboxylation experiment using benzoylformate as the substrate, and a small amount of benzaldehyde was detected with a relatively long time of reaction (10 h). Benzoylformate is a middle metabolite of the phenylalanine metabolism pathway, which is present in *A. pasteurianus*. Phenylalanine can be converted to phenylpyruvate by aspartate aminotransferase and then decarboxylated to phenylacetaldehyde by decarboxylase. Phenylacetaldehyde dehydrogenase converts phenylacetaldehyde to phenylacetate, which is then converted to phenylacetyl-CoA by acyl-CoA thioesterase. Phenylacetyl-CoA can be converted to phenylglyoxylyl-CoA and benzoylformate. Phenylalanine is abundant in vinegars [25], and benzaldehyde is another kind of major volatile compound in Chinese vinegars [6]. Therefore, AlsS2 is likely to make a contribution to benzaldehyde generation in vinegars. Although AlsS2 cannot catalyze the decarboxylation of pyruvate to form acetoin in vitro, its deletion did affect acetoin biosynthesis in *A. pasteurianus*. Given that ALS and BFD can catalyze both the decarboxylation and carboligation reactions, AlsS2 may use pyruvate as the substrate and form acetoin in *A. pasteurianus* cells.

The activity of many genes is determined by the presence of selected substances in the culture media. Because glucose in the culture medium did not result in obvious acetoin generation in *A. pasteurianus*, we used lactic acid as the carbon source in the culture medium to analyze acetoin generation. When *A. pasteurianus* CICC 22518 was cultured in LYSE medium, metabolite analyses revealed that acetoin started to generate at the early logarithmic phase and reached the maximal concentration (about 3.1 g/L) at the late logarithmic phase when lactic acid was almost depleted (Figure 6), suggesting that acetoin was an important metabolite of lactic acid catabolism in *A. pasteurianus*. The excreting of acetoin was regarded as a carbon-overflow metabolic pathway in most known acetoin-producing species. In other words, when excessive carbon source (e.g., glucose and sucrose) was supplemented, the genes of ALS and ALDC would be transcribed and expressed. However, in this study, not glucose but lactic acid supplementation resulted in obvious acetoin generation in *A. pasteurianus*, indicating that the activities of the acetoin biosynthesis genes were related to lactic acid but not glucose in *A. pasteurianus*. These two *alsS*-defective mutants also had a suitable capacity of assimilating lactic acid, but the assimilation rates were reduced in comparison with the wild strain. It was presumed that the coexistence of these two *alsS* genes in tandem could help cells rapidly and securely convert lactic acid into neutral metabolite acetoin so as to deal with the lactic acid pressure in the extracellular environment because there was a large amount of lactic acid in vinegar *Pei* [26]. The *alsS1*-defective mutant was dramatically attenuated in the ability to generate acetoin, but only a 21.6% decrease in acetoin yield was detected in the *alsS2*-defective mutant. AlsS1 seemed to represent the major ALS and play an indispensable role in acetoin biosynthesis in *A. pasteurianus*.

About 71.6% of lactic acid can be converted to acetoin in the wild strain CICC 22518, indicating that the flux from lactic acid to acetoin is the main pathway in *A. pasteurianus*. Several genes potentially coding for the lactate use proteins and lactate dehydrogenases were detected in the chromosomal sequences of *A. pasteurianus* CICC 22518. These enzymes can convert lactic acid to pyruvate, and then ALSs and ALDC can catalyze the decarboxylation reactions of pyruvate and subsequent α-acetolactate to produce acetoin. When the concentration of lactic acid was lower than about 1 g/L, acetoin started to be decomposed as a substitutional carbon source. The *acoABC* gene cluster responsible for acetoin degradation was also detected in the chromosomal sequences, indicating that the other physiological meaning of the lactate-to-acetoin pathway was a novel carbon source use strategy in *A. pasteurianus*.

The *alsR* gene is recognized as a positive regulator gene for the structural genes in acetoin operons [21]. In the *slaR*-defective mutant of *S. marcescens* MG1, acetoin was undetectable in the culture broths, and the transcription products of the genes coding for ALS and ALDC were not detected [27], indicating that this LysR-type regulator was critical for the expression of the acetoin operon in *S. marcescens*. In this study, only 28.1% acetoin decline and 22.4–24.3% decrease in *alsDS1* transcription were detected in the *alsR*-defective mutant in comparison with the wild strain and the *alsS2* gene transcription was not affected. It was presumed that the *alsR* gene was the positive regulator of *alsD* and *alsS1*, but it did not regulate *alsS2*. In addition to the *alsR* gene, other regulator genes may also participate in the regulation of the acetoin operon of *A. pasteurianus*, and 16 genes were annotated as “LysR family transcriptional regulator” in the chromosome of *A. pasteurianus* CICC 22518. In addition, no obvious transcriptional terminator was detected between *alsS1* and *alsS2*. Promoter analysis was carried out for the flanking regions between *alsS1* and *alsS2* using the Prodoric Virtual Footprint website [28], and the *oxyR* binding sites were likely present. The *alsS2* gene may be regulated by other regulators, and its presence may provide additional insurance for the acetoin generation to maintain the intracellular acid balance in *A. pasteurianus*.

In addition, vinegar is an important condiment throughout the world, but people from different geographic regions have different opinions toward the preference of acetoin in vinegars. In Japan, acetoin is regarded as an unfavorable volatile compound in rice vinegar, so some bioengineering techniques are performed to change the acetoin metabolism in AAB strains to reduce acetoin content in vinegars [19]. *A. pasteurianus* is also widely applied in industrial vinegar production in Japan. Therefore, the *alsS*- and *alsR*-defective mutants in this study could be applied in Japanese rice vinegar production to reduce the acetoin yield.

## 5. Conclusions

This is the first identification of two *alsS* genes in tandem involved in acetoin biosynthesis in bacteria. Nevertheless, they did not contribute equally to the acetoin production process. The *alsS1* gene took a major role, and the *alsS2* gene had an auxiliary effect. The *alsR* gene can partly affect the transcriptions of *alsD* and *alsS1* so as to integrate the acetoin biosynthesis regulation, but the *alsS2* gene was not regulated by *alsR*.

## Figures and Tables

**Table 1:** The bacterial strains and plasmids used in this study.

Strains and Plasmids	Genotype or Properties ^1^
Strains	
*Acetobacter pasteurianus* CICC 22518	Wild strain, Cm^R^
*A. pasteurianus* 22518-ΔalsS1	The *alsS1*-defective mutant of *A. pasteurianus* CICC 22518
*A. pasteurianus* 22518-ΔalsS2	The *alsS2*-defective mutant of *A. pasteurianus* CICC 22518
*A. pasteurianus* 22518-ΔalsR	The *alsR*-defective mutant of *A. pasteurianus* CICC 22518
*Escherichia coli* DH5α	F^−^, φ80d/*lacZ* ΔM15, Δ(*lacZYA*-*argF*) U169, *deoR*, *recA1*, *endA1*, *hsdR17*(*rk^−^*, *mk^+^*), *phoA*, *supE44*, *λ^−^*, *thi-1*, *gyrA96*, *relA1*
*E. coli* BL21(DE3)	F^−^, *ompT*, *hsdS*_B_ (r_B_*^−^*m_B_*^−^*), *gal* (λ *cl857*, *ind1*, *sam7*, *nin5*, *lacUV5*-T*7gene1*), *dcm* (DE3)
*E. coli* HB101(pRK2013)	*E. coli* HB101 containing pRK2013 plasmid
*E. coli* BL21-S1	*E. coli* BL21(DE3) containing pET28S1 plasmid, Km^R^
*E. coli* BL21-S2	*E. coli* BL21(DE3) containing pET22S2 plasmid, Amp^R^
Plasmids	
pET-22b(+)	Expression vector, Amp^R^
pET-28a(+)	Expression vector, Km^R^
pET28S1	Expression plasmid based on pET28a(+) for the *alsS1* gene expression, Km^R^
pET22S2	Expression plasmid based on pET22b(+) for the *alsS2* gene expression, Amp^R^
pKOS6b	Gene deletion vector, Km^R^, FC^S^
pKOS6b-ΔalsS1	Gene deletion plasmid based on pKOS6b for the *alsS1* gene deletion, Km^R^, FC^S^
pKOS6b-ΔalsS2	Gene deletion plasmid based on pKOS6b for the *alsS2* gene deletion, Km^R^, FC^S^
pKOS6b-ΔalsR	Gene deletion plasmid based on pKOS6b for the *alsR* gene deletion, Km^R^, FC^S^

^1^ Cm^R^: resistance to chloramphenicol; Km^R^: resistance to kanamycin; Amp^R^: resistance to ampicillin; FC^S^: sensitivity to 5-fluorocytosine.

**Table 2:** The primers used in this study.

Primers	Sequences	Target Genes
28alsS1F	GGGAATTCCATATGACCAATCCGGCAGACAAC	*alsS1*
28alsS1R	CCGCTCGAGTTACGCTGCGGCACTCGTTTCAC	
22alsS2F	GGGAATTCCATATGGCAGTAACATCTGTAGAAAC	*alsS2*
22alsS2R	CCGCTCGAGGCCCGCTGCAATCATAATTTC	
LS1-dup1	TCGAGCTCGGTACCCGGGTCTGCAAAGGGGGCGGTGTC	Upstream of *alsS1*
S1-ddw1	CGCAGCTTTCAGTTCTGTCCTGCAAAAACTG	
S1-dup2	AGGACAGAACTGAAAGCTGCGCAAAAAACGCAGGCCAG	Downstream of *alsS1*
LS1-ddw2	GACGGCCAGTGCCAAGCTTGCCGCATGAAGTTCTTGAGC	
LS2-dup1	TCGAGCTCGGTACCCGGGGCTAAGGTAGACCGTTTGTTTG	Upstream of *alsS2*
S2-ddw1	AGACACTCTACGCCTGCCCCCTATTCTG	
S2-dup2	GGGCAGGCGTAGAGTGTCTGGCTGCAAAAACC	Downstream of *alsS2*
LS2-ddw2	GACGGCCAGTGCCAAGCTTACGGCACCGTAGGACAGACC	
L-R-dup1	TCGAGCTCGGTACCCGGGTCATCACCGCCAGCCTTGAAG	Downstream of *alsR*
L-R-ddw1	ATTACATATACCATCCGCCTCAGCCCTGCTGC	
L-R-dup2	TGAGGCGGATGGTATATGTAATACATATGG	Upstream of *alsR*
L-R-ddw2	GACGGCCAGTGCCAAGCTTGTAGGCGTTATCCAGTTCTGC	
alsDup1	CGCCTTTTGCCTGCGTAAC	*alsD*
alsDdw1	AACACCGTGCGGGTATCAAC	
alsS1up1	CATGGTGGAAATGCAGGAGC	*alsS1*
alsS1dw1	AGGGCCTGACGCACTTCG	
alsS2up1	CTGCGCGGTGTATCAGATGTC	*alsS2*
alsS2dw1	GCCGCCTTTTGCAACATG	
gyrAup1	GTTTGGGCGTCGCTCTTC	*gyrA*
gyrAdw1	TGGCAGCCACTTCTTTCC	

**Table 3:** Effects of metal ions, cofactors, and branched-chain amino acids on AlsS1 activity.

Chemical Agents (Final Concentration)	Relative Activity (%) ^1^
Na^+^ (10 mM)	105.3 ± 9.4
K^+^ (10 mM)	98.9 ± 8.6
EDTA (5 mM)	31.6 ± 0.2
NADP^+^ (10 μM)	100.2 ± 5.0
NADPH (10 μM)	102.3 ± 5.5
NAD^+^ (10 μM)	99.4 ± 7.5
NADH (10 μM)	102.2 ± 4.3
Leucine (10 μM)	100.9 ± 3.9
Isoleucine (10 μM)	95.6 ± 5.7
Valine (10 μM)	106.8 ± 2.3

^1^ values were mean ± standard errors of three replicates.

## Data Availability

The data presented in this study are available on request from the corresponding author.

## Supplementary Materials

The following are available online at https://www.mdpi.com/article/10.3390/foods10051013/s1, Figure S1: GC-FID profile of the reaction mixture in the benzoylformate decarboxylase activity assay.
