# Supplementary material for: Characterization and Regulation of the Acetolactate Synthase Genes Involved in Acetoin Biosynthesis in Acetobacter pasteurianus
Source: Foods. 2021 May 6;10(5):1013. doi: 10.3390/foods10051013 (PMC8148554; doi:10.3390/foods10051013)
Supplement: Supplementary file 1 [file foods-10-01013-s001.zip › foods-1184245-supplementary.pdf]

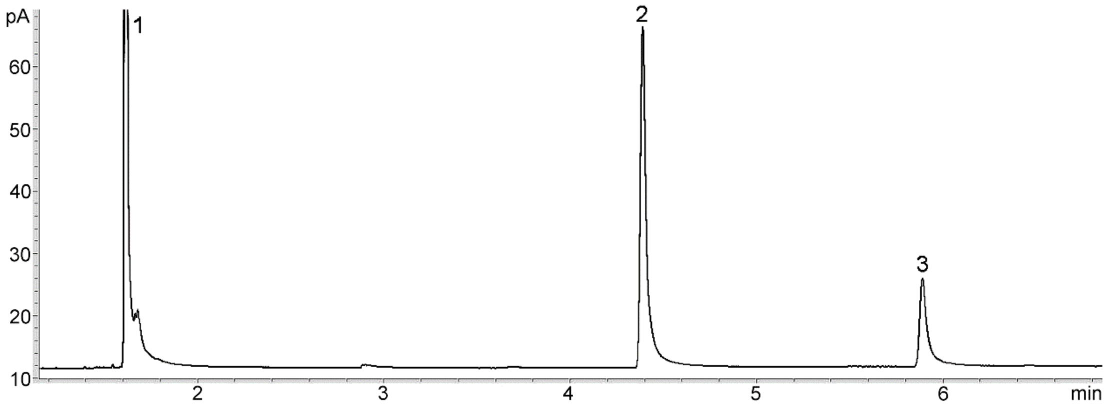

Figure S1. GC-FID profile of the reaction mixture in the benzoylformate decarboxylase activity assay. Peaks: 1, dichloromethane; 2, *n*-hexanol (internal standard); 3, benzaldehyde.
